# Long Non-coding RNA MEG3 Promotes Pyroptosis in Testicular Ischemia-Reperfusion Injury by Targeting MiR-29a to Modulate PTEN Expression

**DOI:** 10.3389/fcell.2021.671613

**Published:** 2021-06-18

**Authors:** Jin-zhuo Ning, Kai-xiang He, Fan Cheng, Wei Li, Wei-min Yu, Hao-yong Li, Ting Rao, Yuan Ruan

**Affiliations:** ^1^Department of Urology, Renmin Hospital of Wuhan University, Wuhan, China; ^2^Department of Anesthesiology, Renmin Hospital of Wuhan University, Wuhan, China

**Keywords:** testicular ischemia-reperfusion injury, MEG3, miR-29, PTEN, pyroptosis

## Abstract

Increasing evidence shows that the abnormal long non-coding RNAs (lncRNAs) expression is closely related to ischemia-reperfusion injury (I/R) progression. Studies have previously described that lncRNA MEG3 regulates pyroptosis in various organs I/R. Nevertheless, the related mechanisms of MEG3 in testicular I/R has not been clarified. The aim of this research is to unravel underlying mechanisms of the regulation of pyroptosis mediated by MEG3 during testicular I/R. We have established a testicular torsion/detorsion (T/D) model and an oxygen-glucose deprivation/reperfusion (OGD/R)-treated spermatogenic cell model. Testicular ischemic injury was assessed by H&E staining. Western blotting, quantitative real-time PCR, MDA, and SOD tests and immunohistochemistry measured the expression of MEG3 and related proteins and the level of ROS production in testicular tissues. Quantitative real-time PCR and western blotting determined the relative expression of MEG3, miR-29a, and relevant proteins in GC-1. Cell viability and cytotoxicity were measured by CCK-8 and LDH assays. Secretion and expression levels of inflammatory proteins were determined by ELISA, immunofluorescence and western blotting. The interaction among MEG3, miR-29a, and PTEN was validated through a dual luciferase reporter assay and Ago2-RIP. In this research, we identified that MEG3 was upregulated in animal specimens and GC-1. In loss of function or gain of function assays, we verified that MEG3 could promote pyroptosis. Furthermore, we found that MEG3 negatively regulated miR-29a expression at the posttranscriptional level and promoted PTEN expression, and further promoted pyroptosis. Therefore, we explored the interaction among MEG3, miR-29a and PTEN and found that MEG3 directly targeted miR-29a, and miR-29a targeted PTEN. Overexpression of miR-29a effectively eliminated the upregulation of PTEN induced by MEG3, indicating that MEG3 regulates PTEN expression by targeting miR-29a. In summary, our research indicates that MEG3 contributes to pyroptosis by regulating miR-29a and PTEN during testicular I/R, indicating that MEG3 may be a potential therapeutic target in testicular torsion.

## Introduction

Testicular torsion is a common emergency in urology department. Due to the rotation of testicular vascular pedicle, testicular necrosis, spermatogenesis disorder and testicular injury are caused, and even testicular resection is inevitable (Mansbach et al., 2005; Al-Maghrebi et al., 2010). Long-term ischemia of testis can cause irreversible damage, so it is necessary to seek treatment quickly and recover blood flow in appropriate time (Turner et al., 2004). However, testicular torsion can lead to progressive reperfusion injury, which is more severe than ischemic injury (Meštrović et al., 2014). The pathological process of testicular torsion exhibits many characteristics of ischemia-reperfusion injury (I/R). Thus, testicular torsion/detorsion (T/D) is thought to be a consequence of I/R. In order to mitigate testicular I/R damage, it is crucial to identify the potential mechanism of testicular I/R development and to identify new and effective molecular biomarkers.

Long non-coding RNAs (lncRNAs) are a type of non-coding RNA molecules that are more than 200 nucleotide units in length (Yan et al., 2017; Ren and Yang, 2018). In recent years, researches have shown that lncRNAs take on the role of competitive endogenous RNAs (ceRNAs) (Kartha and Subramanian, 2014), namely, as miRNA “sponges,” may reduce the expression of miRNAs, and then control the inhibition of miRNAs (Zhang et al., 2012; Wei et al., 2018). More and more studies have confirmed that lncRNAs, as a new class of regulatory factors, appear in biological and pathological processes including heart disease (Cheng et al., 2018), myocardial infarction (Wang et al., 2014), stroke (Bao et al., 2018), obesity (Urkmez et al., 2018), and tumor development (Chen X. et al., 2019; Chen Z. et al., 2019). Thus, in the process of I/R lncRNAs may have tremendous potential as a new therapeutic target.

lncRNA maternally expressed gene 3 (MEG3) is localized on human chromosome 1432.3 and can be widely expressed in normal human tissues, with decreased expression in some tumor cells, and MEG3 overexpression can inhibit tumor cell proliferation and migration (Shihabudeen Haider Ali et al., 2019; Benetatos et al., 2011). Recent researches have shown that MEG3 plays a crucial role in of a variety of organ I/Rs progress. For instance, Liang et al. demonstrated that lncRNA MEG3 increased pyroptosis by targeting miR-29a/AIM2 signaling pathway, thus promoting cerebral I/R (Liang et al., 2020). Huang et al. reported that lncRNA MEG3 mediates hepatic I/R through the miR-34a/Nrf2 axis (Huang et al., 2018). Zou et al. (2019) confirmed the role of MEG3 in myocardial I/R by inhibiting the expression of miR-7-5p. Nevertheless, the biological effect of MEG3 in testicular I/R has not been elucidated. We conjectured that lncRNA MEG3 could be a new biomarker associated with testicular I/R progression.

Pyroptosis is a specific form of inflammatory cell death, which is characterized by cell membrane rupture, elevated expression of key pyroptosis proteins such as NLRP3 and Caspase-1, and release of inflammatory factors such as L-1B and L-18. Caspase-1-mediated pyroptosis is the classical pyroptosis pathway, and Caspase-1 activation is dependent on NLRP3 inflammasome (Malik and Kanneganti, 2017). NLRP3 inflammasomes are inflammatory protein complexes that can be activated by various damage-associated molecular patterns (DAMP) or pathogen-associated molecular patterns (PAMP) (Malik and Kanneganti, 2017). Recent studies have shown elevated expression of NLRP3 in a variety of testicular diseases (Minutoli et al., 2015; Fouad et al., 2020). Thus, pyroptosis mediated by NLRP3 inflammasomes may play a key role in the testicular I/R process.

This study proved that MEG3 had a crucial effect on regulating testicular I/R-induced pyroptosis, and unraveled its regulatory role in the MEG3/miR-29a/PTEN axis. Therefore, the results provide novel insight into testicular I/R.

## Materials and Methods

### Experimental Animals and I/R Model

Male C57BL/6 mice (7–10 week of age) weighing 20–25 g were obtained from the Hubei Provincial Center for Disease Control and Prevention. All mice were randomly divided into 5 groups of 6 mice each. All steps of this experiment were approved by the Wuhan University Animal Care and Use Committee and were performed in accordance with the NIH Guide for the Care and Use of Laboratory Animals. Mice could unrestricted get water and food prior to the experiment. Prior to modeling, we anesthetized mice by intraperitoneal injection of 10% chloral hydrate (0.4 ml/100 g) into the mice. Next we rotated the left testis 720 degrees clockwise and attached it to the scrotal skin with 5/0 silk thread after anesthesia. After 1 h, the twisted testis was then restored to its normal position and the animals were reperfused for 0, 4, 8, or 16 h. Then we anesthetized the mice again, removed and preserved the left testis of each mouse quickly. In addition, we injected adenoviral vectors with si-MEG3 and its negative control (si-NC) into the vas deferens of mice (6 per group) under anesthesia.

### Cell Culture and Treatment

We purchased GC-1 spermatogenic cells from ATCC (American Typical Culture Collection, Manassas, VA, United States) and cultured them in Dulbecco’s Modified Eagle Medium (DMEM; Gibco) under normal oxygen conditions. We replaced glucose-containing DMEM medium with glucose-free DMEM medium and maintained the cells under hypoxic conditions for 3 h to simulate OGD/R (oxygen-glucose deprivation/reoxygenation) injury *in vitro*. Next, the cells were then switched to glucose-containing medium and incubated under normal oxygen conditions for 0, 6, 12, or 24 h. In addition, in the NLPR3 inhibition experiment, 50 μM MCC950 (MedChem Express, Shanghai, China) dissolved in dimethylsulfoxide was given in GC-1 cells 1 h before OGD/R treatment.

### Cell Transfection

Short hairpin RNAs (shRNAs) targeting MEG3, miR-29a mimics, miR-29a inhibitors, ad-PTEN, si-PTEN, and the corresponding negative controls were purchased from Biosci Biotechnology (Wuhan, China). We cloned full-length MEG3 into the pcDNA3.1 vector and performed cell transfection using Lipofectamine 2000 transfection reagent (Invitrogen Life Technologies, Carlsbad, CA, United States) according to the manufacturer’s instructions.

### H&E Staining

After the operation, we fixed the testicular tissue with 4% paraformaldehyde and partially inserted it in paraffin. Finally, 5-μm testicular sections were observed under the microscope after staining with hematoxylin-eosin (H&E).

### RNA Isolation and qRT-PCR

Total RNA was extracted from cells and testicular tissue using TRIzol reagent (Invitrogen Life Technologies, United States) according to the manufacturer’s instructions. Total cellular RNA was reverse transcribed into cDNA using a reverse transcription kit (Taka Biotechnology, Dalian, China). Real-time polymerase chain reaction with the 7900 High Temperature Real-Time Polymerase Chain Reaction System (Applied Biosystems) with GAPDH and as an internal control. The relative expression of miR-29a, PTEN, NLRP3, and caspase-1 was normalized to snRNA U6 (for miRNAs) or GAPDH (for mRNAs). In addition, used 2−ΔΔCt method to determine the level of expression of target genes.

### Measurement of ROS Production

GC-1 cells were incubated in dihydroethidium (DHE, S0063; Beyotime) for 30 min at room temperature in a light-free environment, and fluorescence was detected by fluorescence microscopy (Olympus) after washing to measure ROS production in the cells. In addition, we measured ROS production by detecting malondialdehyde (MDA, S0131; Beyotime) and superoxide dismutase (SOD, S0101; Beyotime) according to the manufacturer’s instructions.

### LDH Release and ELISA Assay

To determine cytotoxicity, we measured the concentration of lactate dehydrogenase (LDH) in the cell supernatant according to the manufacturer’s instructions (Clontech, Mountain View, CA, United States). In addition, we measured the protein concentrations of IL-1β and IL-18 in the culture medium supernatant or serum by mouse ELISA kits (Thermo Fisher Scientific).

### Western Blot Analysis

GC-1 cells and testicular tissue were lysed on ice with RIPA lysis solution (P0013B, Beyotime) and then proteins were extracted. Samples were transferred to nitrocellulose membrane after electrophoresis on 12% SDS-PAGE, and the membranes were treated at 4°C with a mixture of antibodies against PTEN (ab267787; Abcam, Cambridge, United Kingdom), Cleaved Caspase-1 (AF4005; Affinity. United States), and NLRP3 (ab263899; Abcam, Cambridge, United Kingdom). After three washes with PBS-Tween 20, horseradish peroxidase-coupled secondary antibodies were added. These signals were detected by ECL (Pierce Biotechnology, Beijing, China) and qualified using ImageJ software (NIH, Bethesda, MD, United States).

### Cell Viability

We used CCK-8 cell viability assay kit (Jiangsu Beyotime) to detect the viability of GC-1 cells. Cells were seeded in 96-well plates and stained with 20 μL CCK-8 reagent for 4 h after 24 h of transfection. Cell viability was detected by spectrophotometer (Thermo Fisher Scientific, Multiskan FC, United States) based on the absorbance at 450 nm. Each test was performed three times.

### Immunofluorescence

We fixed cultured GC-1 cells in 4% paraformaldehyde buffer in PBS. The cells were then penetrated and incubated with a blocking solution for 2 h at room temperature and then incubated overnight at 4°C with anti-NLRP3 and anti-caspase-1 antibodies. Thereafter, cells were incubated with the corresponding secondary antibody (Thermo Fisher Scientific) for 1 h at room temperature. Then the nuclei were stained with 4′, 6-diamidino-2phenylindole (DAPI, Thermo Fisher Scientific) and left at room temperature for 20 min. Finally, immunofluorescence was examined under a fluorescence microscope.

### Dual Luciferase Reporter Assay and RNA Immunoprecipitation (RIP) Assay

We verified the direct interaction between MEG3, miR-29a, and PTEN by dual luciferase reporter assay and Ago2-RIP. For detailed steps refer to our previous study (Li et al., 2018). Transfection procedures and measurements were as previously described.

### Immunohistochemistry

We detected the expression of PTEN, NLRP3, and caspase-1 by immunohistochemistry. Fixed testicular tissue in 4% paraformaldehyde and made into sections. Incubations were performed using primary antibodies against PTEN (ab267787; Abcam, Cambridge, United Kingdom), cleaved Caspase-1 (AF4005; Affinity, United States) and NLRP3 (ab263899; Abcam, Cambridge, United Kingdom), and all sections were analyzed under microscopic examination.

### Statistical Analysis

All experiments were performed at least 3 times. Data are expressed as the average ± the standard deviation. Comparisons between two groups were made using a two-tailed Student’s *t*-test, and multiple comparisons were made using one-way ANOVA. SPSS 17.0 was used for statistical analysis and *P* < 0.05 was regarded as statistically significant.

## Results

### MEG3 Is Upregulated in Testicular I/R and Cellular OGD/R

**FIGURE 1:**
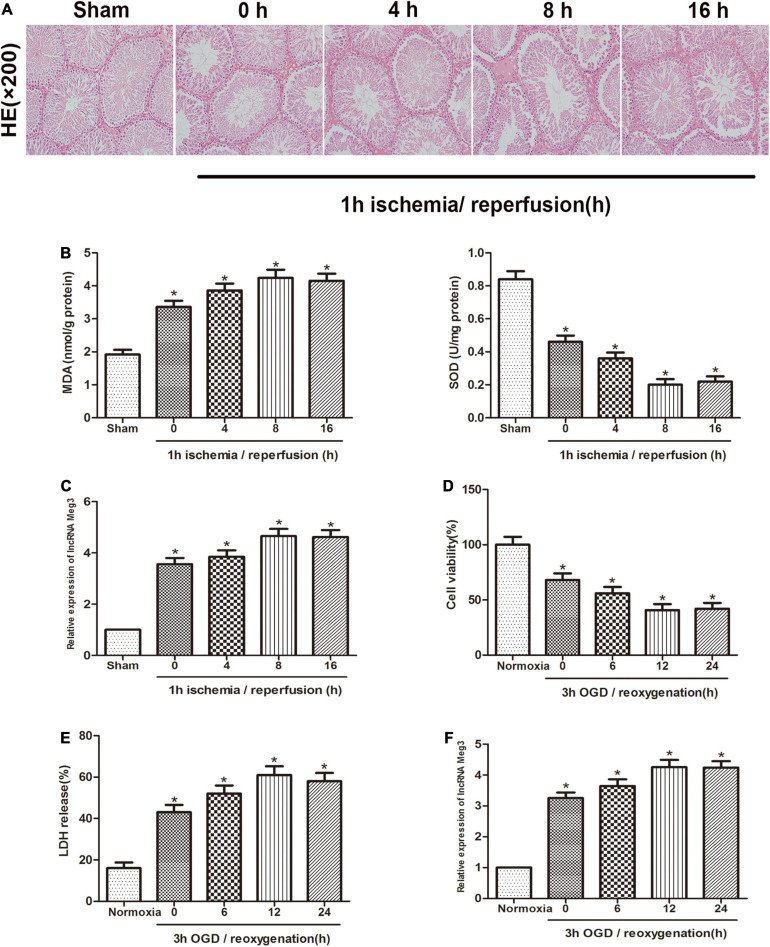
MEG3 is upregulated in testicular I/R and cellular OGD/R. **(A)** Comparison of testicular H&E staining in the sham-operated group and at different reperfusion times (0, 4, 8, and 16 h) after 1 h of ischemia. **(B)** Comparison of MDA and SOD levels in the sham-operated group and in animal tissues at different reperfusion times (0, 4, 8, and 16 h) after 1 h of ischemia. **(C)** Comparison of MEG3 expression levels by qRT-PCR in sham-operated group and animal samples at different reperfusion times (0, 4, 8, and 16 h) after 1 h of ischemia. **(D)** Comparison of cell viability between normoxic group and GC-1 cells at different reoxygenation times (0, 6, 12, and 24 h) after 3 h of OGD treatment by CCK-8 assay. **(E)** Comparison of LDH release in normoxic group and cells at different reoxygenation times (0, 6, 12, and 24 h) after 3 h of OGD treated. **(F)** Comparison of relative expression levels of MEG3 in normoxic group and cells at different reoxygenation times (0, 6, 12, and 24 h) after 3 h of OGD treated. The data are presented as the mean ± S.E.M. *n* = 6. *p < 0.05 vs. control.

First, we established a mouse ischemia-reperfusion model to identify the effect of lncRNA-MEG3 in the testicular I/R process. Specimens from animals that were reperfused for 0, 4, 8, or 16 h after 1 h of ischemia were subjected to H&E staining and MDA and SOD detection (Figures 1A,B). The results showed that the function of testicular spermatogenic cells was severely impaired, and compared with that in the sham group, the cellular reactive oxygen content in I/R group gradually increased with the increasing reperfusion time and reached a peak at 8 h. These results indicate that we successfully established a testicular I/R model. Then, we detected MEG3 expression in mouse testis tissue by qRT-PCR, and the analysis showed that MEG3 levels gradually increased as the reperfusion time increased and reached the highest value at 8 h (Figure 1C). In addition, we used mouse GC-1 cells to further study MEG3 expression in the cellular OGD/R model. The CCK-8 experiment indicated that GC-1 cells viability was decreased after OGD/R treatment, especially in cells subjected to 3 h of OGD/12 h of reoxygenation, and the cell viability of the OGD/R group continued to drop to approximately 40%, which was significantly different from that of the normoxia group (Figure 1D). Furthermore, LDH release test data showed that OGD/R increased the release of LDH from cells, and the LDH release of the OGD/R group continued to rise to 60% at h of OGD/12 h of reoxygenation, which was significantly different from that of the normoxia group (Figure 1E). In addition, the expression of MEG3 was significantly increased in the OGD/R group after 3 h of OGD/12 h of reoxygenation compared with that in the normoxia group (Figure 1F). This is consistent with the decreasing trend of the cell survival rate and the increasing trend of LDH release and is also in line with the trend observed in the I/R model in mice. In summary, these experimental results suggest that MEG3 may be involved in the testicular I/R progression.

### Influence of lncRNA MEG3 on Pyroptosis and Inflammation of GC-1 Cells *in vitro*

**FIGURE 2:**
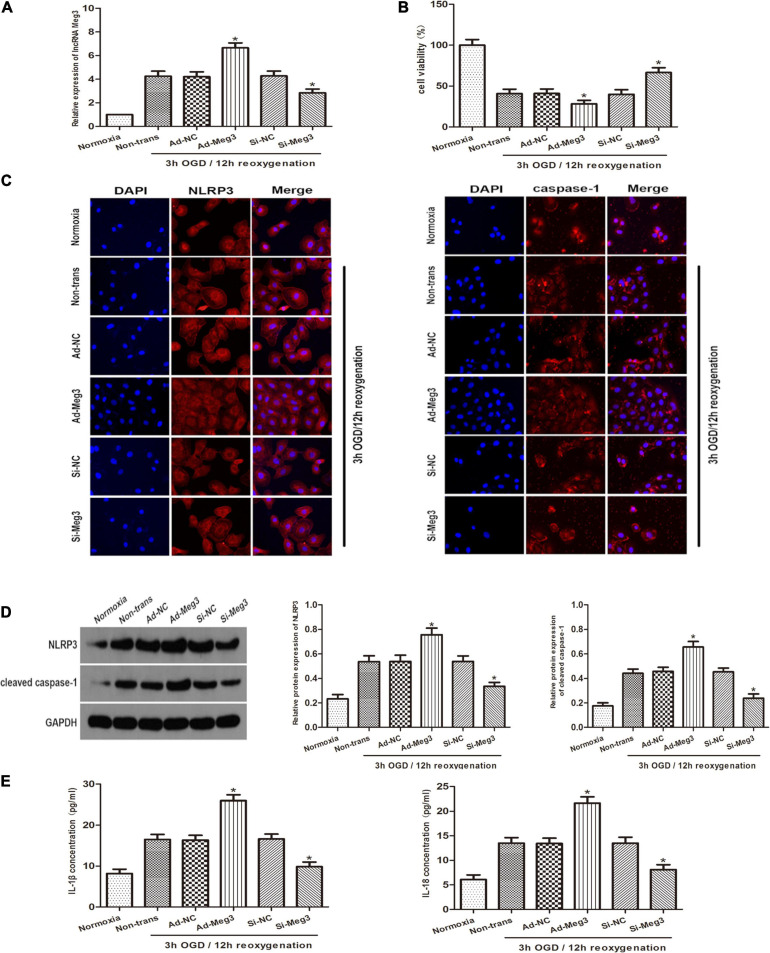
Influence of lncRNA MEG3 on pyroptosis and inflammation of GC-1 cells *in vitro*. **(A)** Comparison of relative expression levels of MEG3 in cells transfected with ad-MEG3 or si-MEG3. **(B)** Cell viability were compared by CCK-8 between NC group cells and cells transfected with ad-MEG3 or si-MEG3 under normoxia or 3 h/12 h OGD/R treatment. **(C)** Expression levels of NLRP3 and caspase-1 were compared by immunofluorescence between NC group cells and cells transfected with ad-MEG3 or si-MEG3 under normoxia or 3 h/12 h OGD/R treatment. **(D)** Cleaved caspase-1 and NLRP3 protein levels were compared by Western blot between NC group cells and cells transfected with ad-MEG3 or si-MEG3 analysis under normoxia or 3 h/12 h OGD/R treatment. **(E)** IL-1β and IL-18 expression were compared by ELISA analysis between NC group cells and cells transfected with ad-MEG3 or si-MEG3 under normoxia or 3 h/12 h OGD/R treatment. The data are presented as the mean ± S.E.M. *n* = 6. *p < 0.05 vs. control.

To explore the role of lncRNA-MEG3 in cell injury caused by OGD, we improved MEG3 expression by transfection with recombinant adenovirus and inhibited MEG3 expression by transfection of GC-1 cells with small interfering RNA (siRNA). qRT PCR analysis of the results showed that in comparison with negative control cells, the expression of MEG3 in cells transfected with ad-MEG3 was significantly increased at 3 h of OGD/12 h of reoxygenation and significantly increased MEG3 expression in cells transfected with si-MEG3 and significantly decreased MEG3 expression in cells transfected with si-MEG3 (Figure 2A). Furthermore, the results of CCK-8 assay showed that overexpression of MEG3 significantly inhibited cell viability at 3 h of OGD/12 h of reoxygenation. However, knockdown of MEG3 led to the opposite result (Figure 2B). Immunofluorescence data showed that in GC-1 cells, MEG3 overexpression significantly promoted NLRP3 and caspase-1 expression, indicating that it promoted epiphora, while silencing MEG3 significantly inhibited epiphora (Figure 2C). Furthermore, western blot analysis showed that MEG3 overexpression increased NLRP3 and caspase-1 expression. Consistently, inhibition of MEG3 expression in GC-1 cells decreased the levels of NLRP3 and caspase-1 (Figure 2D). ELISA analysis showed that MEG3 overexpression increased IL-1β and IL-18 levels. Consistently, inhibition of MEG3 expression in GC-1 cells decreased IL-1β and IL-18 levels. Collectively, these results suggest that MEG3 overexpression further promotes epithelial pyroptosis in testicular spermatogenic cells (Figure 2E).

### LncRNA MEG3 Inhibits miR-29a Expression by Directly Targeting It

**FIGURE 3:**
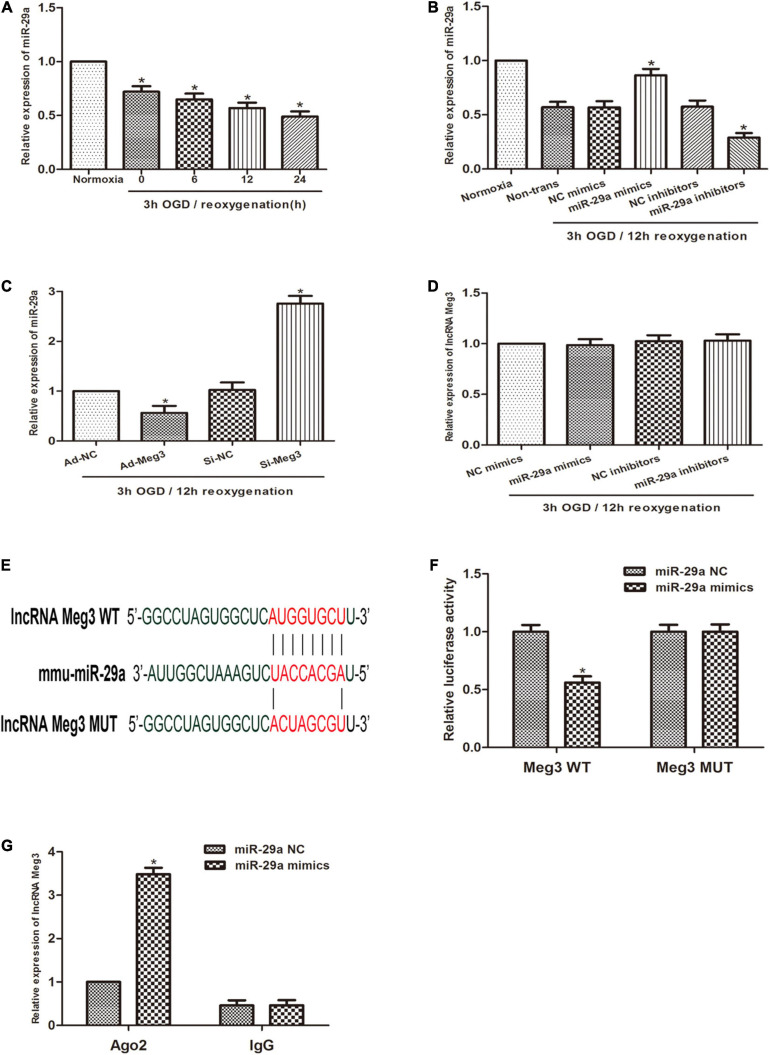
lncRNA MEG3 inhibits miR-29a expression by directly targeting it. **(A)** Comparison of the relative expression levels of miR-29a between normoxic group and GC-1 cells at different reoxygenation times (0, 6, 12, and 24 h) after 3 h of OGD treatment. **(B)** Comparison of the expression level of miR-29a in cells transfected with miR-29a mimic or miR-29a inhibitor by qRT-PCR under normoxia or 3 h/12 h OGD/R treatment. **(C)** Comparison of the relative expression of MEG3 in cells transfected with ad-MEG3 and si-MEG3 by qRT-PCR under 3 h/12 h OGD/R treatment. **(D)** Comparison of the relative expression of miR-29a in cells transfected with miR-29a mimic or miR-29a inhibitor by qRT-PCR under 3 h/12 h OGD/R treatment. **(E)** Prediction of MEG3 binding sites to miR-29a. **(F)** A dual-luciferase reporter assay was executed to measure luciferase activity in cells cotransfected with miR-29a mimic and MEG3-WT or MEG3-Mut. **(G)** Comparison of MEG3 expression levels by RIP assay and qRT-PCR in cells transfected with miR-29a mimics or miR-29a NC. The data are presented as the mean ± S.E.M. *n* = 6. *p < 0.05 vs. control.

Previous studies reported that miR-29a can inhibit oxidative stress and the pathway of NLRP3-mediated pyroptosis and has a protective effect on myocardial I/R. Therefore, we sought to describe the interaction of MEG3 and miR-29a in testicular I/R. First, qRT-PCR profiling revealed that miR-29a expression was lower in the OGD/R-induced cell injury group than in the normoxia group (Figure 3A), and miR-29a expression was negatively correlated with MEG3 expression. Next, we used miR-29a mimics and miR-29a inhibitors to transfect GC-1 cells to alter the levels of miR-29a in GC-1 cells and verified the transfection efficiency by qRT-PCR analysis (Figure 3B). To demonstrate that miR-29a is modulated by MEG3, we tested the miR-29a expression levels in GC-1 cells that were transfected with ad-MEG3 and si-MEG3 compared to the expression levels of negative controls. The results revealed that the miR-29a expression was dramatically reduced by the overexpression of MEG3 (Figure 3C). To investigate whether MEG3 is under the regulation of miR-29a, we examined the level of MEG3 expression in GC-1 cells, which were transfected the miR-29a mimics, or miR-29a inhibitors and their corresponding negative controls. However, the results indicated that miR-29a overexpression did not reduce MEG3 expression (Figure 3D). To verify whether lncRNA MEG3 binds directly to miR-29a, we used the LncBase Experimental v2 database to perform *in silico* prediction of the target site of the miR-29a sequence (Figure 3E). The analysis of the dual luciferase reporter showed that cotransfection of miR-29a mimics and MEG3-WT resulted in a significant decrease in luciferase activity compared to the corresponding controls. Nevertheless, MEG3-Mut eliminated the impact of miR-29a mimics on luciferase activity in OGD/R cells (Figure 3F), suggesting that MEG3 interacts directly with miR-29a. To evaluate the interactions between them more directly, we performed Ago2-RIP analysis. The levels of endogenous MEG3, which is resistant to Ago2 pulldown, were significantly enriched in OGD/R cells transfected with miR-29a mimics (Figure 3G). In conclusion, these results suggest that MEG3 serves as a sponge for miR-29a and inhibits its expression.

### PTEN Is the Target Gene of miR-29a and Is Regulated by MEG3, and Regulate Pyroptosis

**FIGURE 4:**
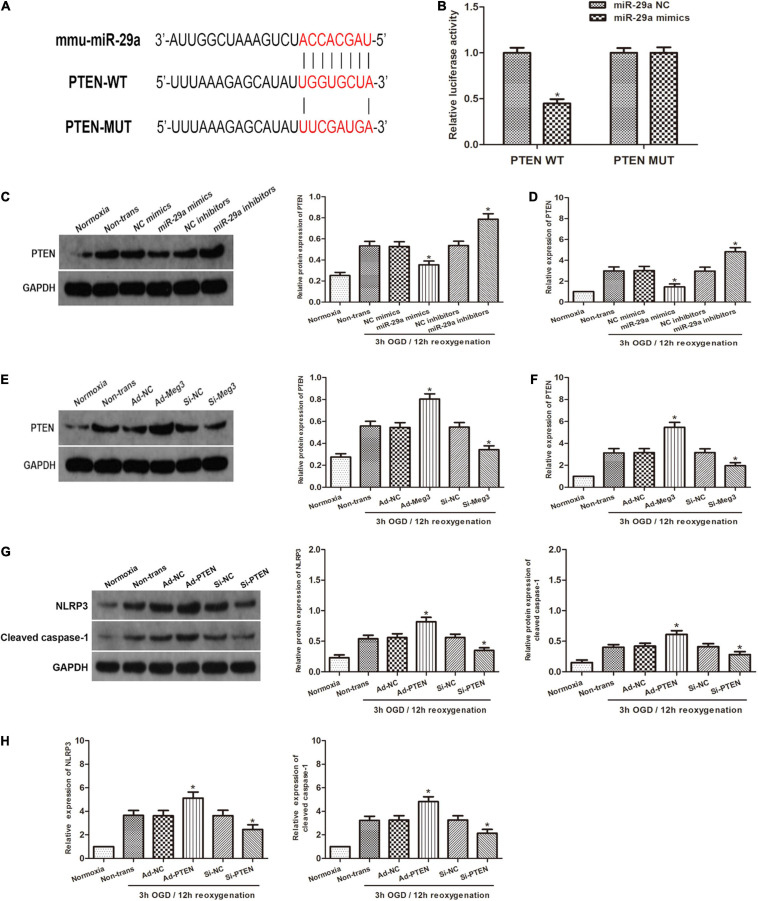
PTEN is the target gene of miR-29a and is regulated by MEG3. **(A)** Prediction of miR-29a binding sites in the 3′UTR of PTEN (PTEN-WT) and its mutant sequence (PTEN-Mut). **(B)** A dual-luciferase reporter assay was executed to measure luciferase activity in cells cotransfected with miR-29a mimic and PTEN-WT or PTEN-Mut. **(C,D)** PTEN protein and mRNA expression of cells transfected with miR-29a mimic or miR-29a inhibitor under normoxia or 3 h/12 h OGD/R treatment. **(E,F)** PTEN protein and mRNA expression of cells transfected with ad-MEG3 or si-MEG3 under normoxia or 3 h/12 h OGD/R treatment. **(G,H)** NLRP3 and cleaved caspase-1 protein and mRNA expression of GC-1 cells transfected with ad-PTEN or si-PTEN under normoxia or 3 h/12 h OGD/R treatment. The data are presented as the mean ± S.E.M. *n* = 6. *p < 0.05 vs. control.

To investigate the relationship between miR-29a and PTEN, we used open software (TargetScan, PicTarget, and miRanda) to make *in silico* predictions. To validate these predictions, we built a luciferase reporter plasmid containing either a PTEN wild type 3′UTR in sequence or a mutant 3′UTR in sequence. The PTEN 3′UTR mutant reporter vector containing the luciferase reporter gene was utilized as a negative control. Examination of the luciferase reporter data showed that overexpression of miR-29a dramatically reduced the activation of the PTEN 3′UTR reporter gene vector, but there was a lack of change in the PTEN-Mut group (Figures 4A,B). To verify the regulatory effect of miR-29a on PTEN, we tested PTEN expression after miR-29a was overexpressed or knocked down. Western blot and qRT-PCR analyses showed that Overexpression of miR-29a decreased PTEN expression, while inhibition of miR-29a expression resulted in a dramatic increase in PTEN expression (Figures 4C,D). To further explore whether MEG3 regulated PTEN, we transfected ad-MEG3 and si-MEG3 to cells and measured PTEN expression. We found that overexpression of MEG3 significantly promoted PTEN expression. On the other hand, MEG3 knockdown resulted in decreased PTEN expression (Figures 4E,F). To investigate whether NLRP3 and caspase-1 are regulated by PTEN, we tested NLRP3 and caspase-1 expression after PTEN was overexpressed or knocked down. Western blot and qRT-PCR analyses displayed that PTEN overexpression significantly promoted NLRP3 and caspase-1 expression, and the inhibition of PTEN expression caused a decrease in NLRP3 and caspase-1 expression (Figures 4G,H). In addition, we used MCC950, a specific inhibitor of NLRP3, to explore that the effect of NLRP3 in this process. Western blot indicated that inhibition of NLRP3 caused a reduction in cleaved caspase-1 expression (Supplementary Figure 1A), and ELISA indicated that inhibition of NLRP3 decreased the levels of IL-1β and IL-18 (Supplementary Figure 1B). These results indicate that the inhibition of NLRP3 can block the pyroptosis in GC-1 cells. In summary, it can be preliminarily concluded that PTEN is a target gene of miR-29a and regulated by MEG3, and regulate pyroptosis through the NLRP3/caspase-1 signaling pathway.

### MEG3 Regulates OGD/R-Induced Pyroptosis and Inflammation

**FIGURE 5:**
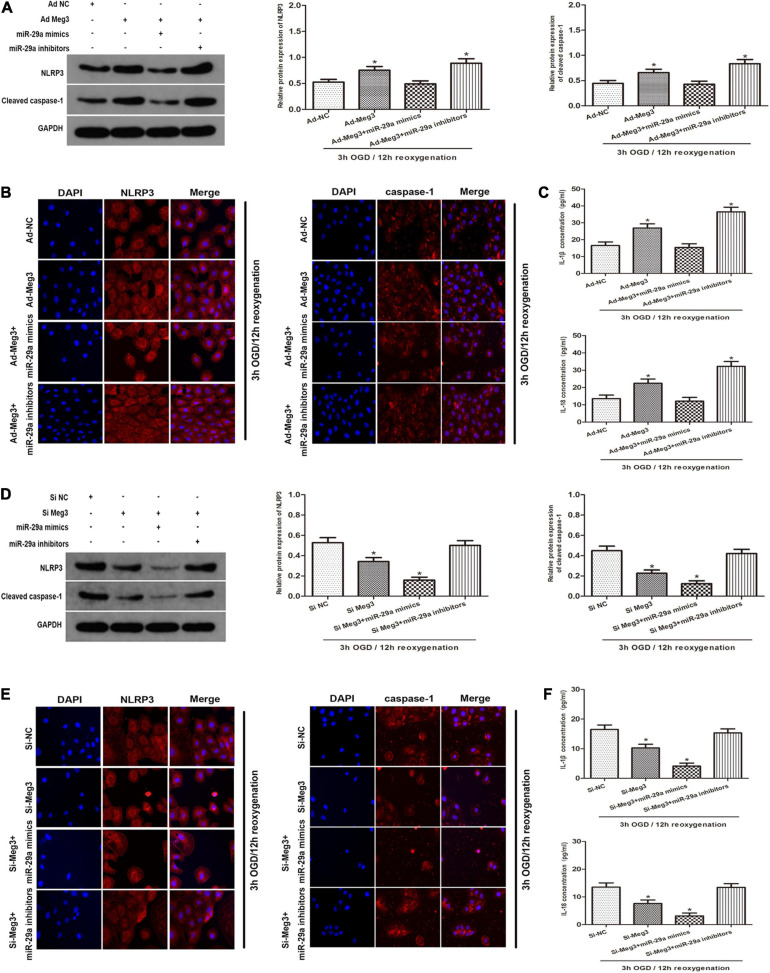
—MEG3 Regulates OGD/R-Induced Pyroptosis and Inflammation. **(A)** MEG3 regulates OGD/R-induced pyroptosis and inflammation. NLRP3 and cleaved caspase-1 protein expression in cells cotransfected with ad-MEG3 and miR-29a mimic or miR-29a inhibitor under 3 h/12 h OGD/R treatment. **(B)** Expression of NLRP3 and caspase-1 was measure by immunofluorescence in GC-1 cells transfected with ad-MEG3 and miR-29a mimic or miR-29a inhibitor under 3 h/12 h OGD/R treatment. **(C)** ELISA analysis was executed to measure the expression of IL-1β and IL-18 in GC-1 cells transfected with ad-MEG3 and miR-29a mimic or miR-29a inhibitor under 3 h/12 h OGD/R treatment. **(D)** NLRP3 and cleaved caspase-1 protein levels in cells cotransfected with si-MEG3 and miR-29a mimic or miR-29a inhibitor under 3 h/12 h OGD/R treatment. **(E)** Expression of NLRP3 and caspase-1 was measured by immunofluorescence in GC-1 cells transfected with si-MEG3 and miR-29a mimic or miR-29a inhibitor under 3 h/12 h OGD/R treatment. **(F)** Measure IL-1β and IL-18 expression by ELISA analysis in cells transfected with si-MEG3 and miR-29a mimic or miR-29a inhibitor under 3 h/12 h OGD/R treatment. The data are presented as the mean ± S.E.M. *n* = 6. *p < 0.05 vs. control.

To clarify the potential mechanism of pyroptosis induced by OGD/R, we tested whether MEG3 regulates the NLRP3, caspase-1, and the inflammatory indicators IL-1β and IL-18. First, we detected the protein levels of the key components that mediate pyroptosis signal transduction by western blotting. The results showed that overexpression of MEG3 after ischemia-reperfusion increased the expression of NLRP3 and cleaved-caspase-1, while the simultaneous transfection of miR-29a mimic counteracted the effect of overexpressed MEG3 on these signaling proteins (Figure 5A). Next, the immunofluorescence staining results showed that the overexpression of MEG3 enhanced the OGD/R-mediated upregulation of NLRP3 and caspase-1, and in the cells cotransfected with ad-MEG3 and miR-29a inhibitor, the fluorescence intensity of NLRP3 and caspase-1 was much more intense than that in the cells transfected with ad-MEG3 alone (Figure 5B). Furthermore, we used ELISA to detect the expression and secretion of IL-1β and IL-18. Compared with the control group, the MEG3 overexpression group exhibited increased the upregulation of IL-1β and IL-18 mediated by OGD/R. The cotransfection of ad-MEG3 and miR-29a mimic partially eliminated the effect of MEG3 overexpression on the IL-1β and IL-18 levels, while the cotransfection of ad-MEG3 and miR-29a inhibitor partially enhanced this effect (Figure 5C). On the other hand, downregulation of MEG3 reduced the expression of NLRP3 and cleaved-caspase-1, while miR-29a inhibitor eliminated the effect of MEG3 downregulation on these signaling proteins (Figures 5D,E). Likewise, the ELISA results had similar implications (Figure 5F). In summary, these data indicate that MEG3 can directly regulate the levels of NLRP3, cleaved caspase-1, IL-1β and IL-18 through miR-29a and can be modulated to regulate OGD/R-induced pyroptosis and inflammation.

### MEG3 Promotes Spermatogenic Cell Pyroptosis Caused by Testicular I/R *in vivo*

**FIGURE 6:**
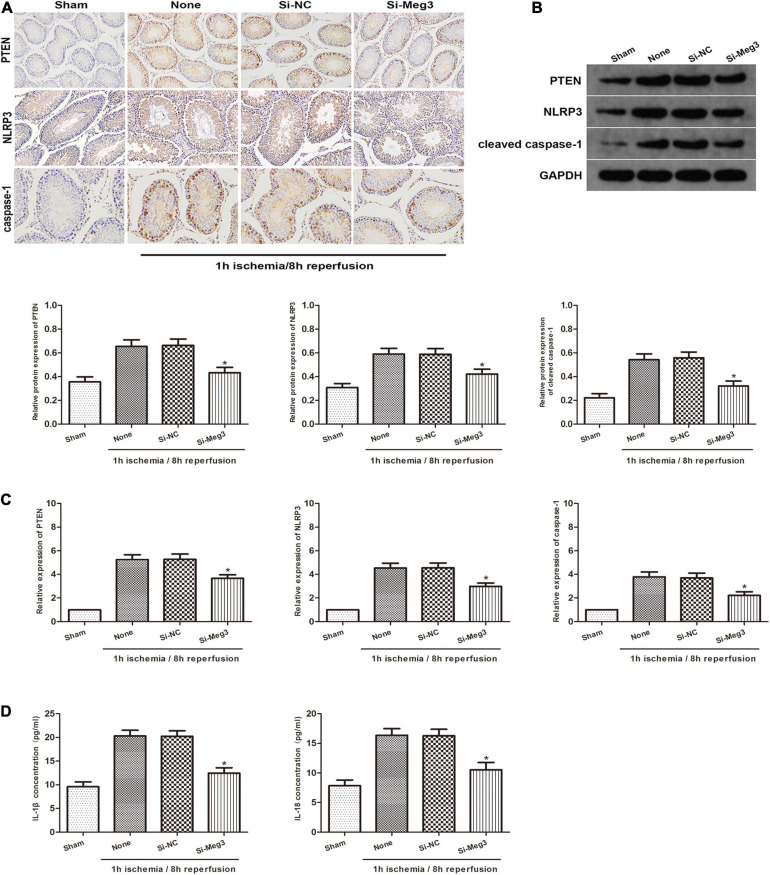
lncRNA MEG3 promotes spermatogenic cell pyroptosis caused by testicular I/R testicular I/R *in vivo*. **(A)** Immunohistochemical staining of PTEN, NLRP3 and caspase-1 in testicular specimen transfected with si-NC or si-MEG3 at 8 h reperfusion after 1 h of ischemia. **(B)** PTEN, NLRP3, and caspase-1 expression in testicular specimen transfected with si-NC or si-MEG3 were detected by western blot at 8 h reperfusion after 1 h of ischemia. **(C)** qRT-PCR was executed to analyze the relative expression of PTEN, NLRP3 and caspase-1 in testicular specimen transfected with si-NC or si-MEG3 at 8 h reperfusion after 1 h of ischemia. **(D)** ELISA analysis was executed to measure IL-1β and IL-18 expression in testicular specimen transfected with si-NC or si-MEG3 at 8 h reperfusion after 1 h of ischemia. The data are presented as the mean ± S.E.M. *n* = 6. *p < 0.05 vs. control.

To identify the role of MEG3 on the pyroptosis of spermatogenic cells *in vivo*, we administered si-MEG3 or si-NC to the vas deferens of mice. We detected PTEN, NLRP3 and caspase-1 expression in testicular specimens by immunohistochemical staining in reaction to 1 h of ischemia/8 h of reperfusion. The findings indicated that the amount of PTEN-, NLRP3- and caspase-1-positive cells in the si-MEG3 group was markedly lower than in the negative control group (Figure 6A). In addition, the western blot indicated that the si-MEG3 treatment reduced PTEN, NLRP3, and cleaved caspase-1 protein levels in si-MEG3 group compared with negative control group (Figure 6B). Second, we analyzed the expression of PTEN, NLRP3 and caspase-1 after si-MEG3 or si-NC injection by qRT-PCR. The results indicated that PTEN, NLRP3, and caspase-1 levels in si-MEG3 group were significantly lower than in control group, which is consistent with the immunohistochemistry results (Figure 6C). Furthermore, ELISA analysis showed that compared with the negative control group, IL-1β and IL-18 expression levels in the si-MEG3 group decreased significantly after MEG3 expression was inhibited (Figure 6D). In summary, these data suggest that lncRNA MEG3 promotes spermatogenic cell pyroptosis in testicular I/R *in vivo*.

## Discussion

Acute torsion of the testis causes local ischemic injury, and reversing the torsion will caused to increased blood flow and consequent reperfusion damage, causing the death of spermatogenic cells and the destruction of reproductive function, which can eventually lead to testicular dysfunction and infertility (Hadziselimovic et al., 1998). The salvage rate of the testis from a manual reduction to a surgical reduction ranges from 42 to 88%(Cattolica et al., 1982; Urkmez et al., 2018), so it is necessary to find an effective treatment for testicular torsion based on the molecular mechanism. The present research shows that lncRNAs play a crucial role in virous organs I/R progress (Lu et al., 2020; Zhou et al., 2020). Thus, it is crucial to elucidate the biological functions of lncRNAs in testicular I/R for better treatment of testicular torsion in the clinical practice.

MEG3 was originally considered a tumor suppressor (Ghafouri-Fard and Taheri, 2019) that plays an important antiproliferative role in cancer cells. Recent studies have shown that MEG3 regulates cell apoptosis by engulfing microribonucleic acids (Moradi et al., 2019), and it is abnormally expressed in the pathological progression of I/R in some organs (Huang et al., 2018; Zou et al., 2019; Liang et al., 2020). Recently, many researches have indicated that pyroptosis may play a crucial role in the control of I/R in various organs (Hua et al., 2019; Ye et al., 2019; Xiao et al., 2020) and that pyroptosis is also closely related to MEG3 (Zhang et al., 2018). Nevertheless, the detailed mechanism of pyroptosis during testicular I/R has not yet been elucidated. We found that MEG3 expression in testicular I/R tissue and OGD/R-treated spermatogenic cells was significantly higher than those in the respective normal controls. High MEG3 expression levels were positively correlated with the degree of pyroptosis of cells both *in vivo* and *in vitro*. In addition, in loss of function or gain of function assays, we demonstrated knocking down MEG3 significantly reduced the level of pyroptosis-related proteins, while overexpression of MEG3 caused the contrary result. This indicates that lncRNA MEG3 may be instrumental in regulating pyroptosis in testicular I/R.

Furthermore, in the present study, we confirmed that miR-29a expression was markedly decreased in the testicular I/R progression and negatively correlated with MEG3 expression. In recent years, there is increasing of evidence that miR-29a has a critical function in ischemia-reperfusion. For example, previous research has demonstrated a role for miR-29a in the progression of myocardial pyroptosis (Ding et al., 2020). MiR-29a has been reported to suppress spermatogenic cells apoptosis in testis I/R by aiming at the TRPV4 pathway (Ning et al., 2017). Our study suggest that miR-29a is regulated down in spermatogenic cells treated with OGD/R., indicating that it has an important role in testicular I/R. Furthermore, we used bioinformatics predictions to identify miR-29a as a possible immediate target downstream of MEG3. To confirm that MEG3 binds to miR-29a in a direct manner, we carried out a luciferase reporter gene test and identified direct binding sites between MEG3 and miR-29a. In conclusion, these data demonstrate that MEG3 regulates miR-29a expression by directly targeting it and has a significant function in testicular I/R.

Phosphatase and Tensin homolog (PTEN) is a constitutively active phosphatase, which is removed on chromosome 10. It is the most commonly removed phosphatase and the second most commonly removed tumor suppressor gene in cancers (Stiles, 2009). A previous study demonstrated that knockout of PTEN inhibited NLRP3-mediated pyroptosis of cardiomyocytes and alleviated myocardial I/R damage (Cui et al., 2020). Recently, there have been many reports that PTEN is a target of miRNAs in a variety of lncRNA-mediated ceRNA networks and has a critical effect on the myocardium, brain cells and renal I/R (Cheng et al., 2019; Ding et al., 2019; Gao et al., 2020). Nevertheless, the association between lncRNA and PTEN in testicular I/R is not yet clarified. This study have found that lncRNA MEG3 exacerbated the heat recession caused by testicular I/R by suppressing the inhibitory effect of miR-29a on PTEN. MEG3 can positively regulate the expression of PTEN in testicular spermatogenic cells by targeting miR-29a. Conversely, overexpression of miR-29a did not alter MEG3 expression but inhibited PTEN upregulation resulting from MEG3 overexpression. In summary, these findings indicate that there is a new MEG3-mediated ceRNA network in testicular I/R and MEG3 can positively regulate PTEN by inhibiting miR-29a, and further regulate pyroptosis.

## Conclusion

In summary, our research shows that the MEG3/miR-29/PTEN axis is participated in the modulation of pyroptosis in testicular I/R. These findings provide the first evidence that during testicular I/R, the MEG3/miR-29/PTEN axis participates in pyroptosis through the NLRP3/caspase-1 signaling pathway. Therefore, this study suggests that MEG3 can be a critical modulator of testicular I/R, and inhibition of MEG3 expression can be a potential therapeutic target for testicular I/R.

## Data Availability Statement

The datasets presented in this study can be found in online repositories. The names of the repository/repositories and accession number(s) can be found in the article/Supplementary Material.

## Ethics Statement

The animal study was reviewed and approved by the Animal Care and Use Committee of Wuhan University.

## Author Contributions

J-zN was responsible for the design of the manuscript’s ideas. K-xH was responsible for the cell and animal experiments and the writing of the manuscript. FC and WL were responsible for the overall ideas, and others were responsible for data processing. All authors contributed to the article and approved the submitted version.

## Conflict of Interest

The authors declare that the research was conducted in the absence of any commercial or financial relationships that could be construed as a potential conflict of interest.

## Supplementary Material

Supplementary Figure 1 The inhibition of NLRP3 can block the pyroptosis in GC-1 **(A)** cleaved caspase-1 protein expression in GC-1 cells with MCC950 treatment in normoxia or with 3 h OGD/12 h reoxygenation treatment. *p<0.05 vs cells without MCC950 treatment, n = 6 per group; **(B)** ELISA analysis was performed to determine the expression of IL-1β and IL-18 in GC-1 cells with MCC950 treatment in normoxia or with 3 h OGD/12 h reoxygenation treatment. *p<0.05 vs. cells without MCC950 treatment, n = 6 per group.
