# Supplementary figures and images for: Long Non-coding RNA MEG3 Promotes Pyroptosis in Testicular Ischemia-Reperfusion Injury by Targeting MiR-29a to Modulate PTEN Expression
Source: Front Cell Dev Biol. 2021 Jun 18;9:671613. doi: 10.3389/fcell.2021.671613 (PMC8249820; doi:10.3389/fcell.2021.671613)

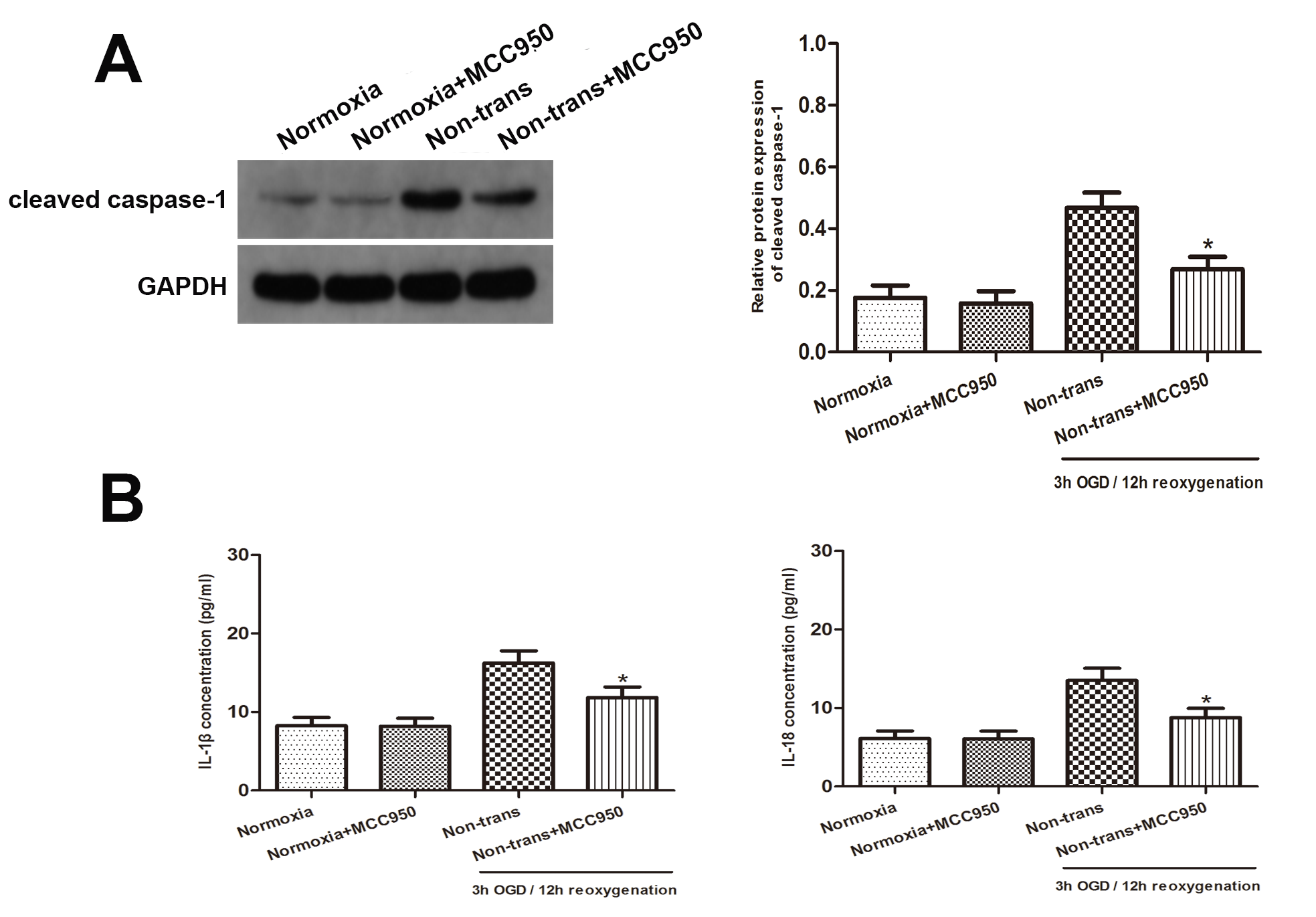

Supplement: Supplementary Figure 1 — The inhibition of NLRP3 can block the pyroptosis in GC-1 (A) cleaved caspase-1 protein expression in GC-1 cells with MCC950 treatment in normoxia or with 3 h OGD/12 h reoxygenation treatment. *p<0.05 vs cells without MCC950 treatment, n = 6 per group; (B) ELISA analysis was performed to determine the expression of IL-1β and IL-18 in GC-1 cells with MCC950 treatment in normoxia or with 3 h OGD/12 h reoxygenation treatment. *p<0.05 vs. cells without MCC950 treatment, n = 6 per group. [file Image_1.TIF]
